# Detection of Hepatitis A RNA, Hepatitis E RNA, Human Adenovirus F DNA, and Norovirus RNA in Fresh and Frozen Berry Products at Point of Retail in Ireland

**DOI:** 10.1007/s12560-023-09561-4

**Published:** 2023-08-01

**Authors:** Charlene Bennett, Kevin Hunt, Francis Butler, Sinead Keaveney, Séamus Fanning, Cillian De Gascun, Suzie Coughlan, Joanne O’Gorman

**Affiliations:** 1 UCD-National Virus Reference Laboratory, University College Dublin, Belfield, Dublin, 4 Ireland; 2 UCD-School of Biosystems and Food Engineering, University College Dublin, Belfield, Dublin, 4 Ireland; 3 Marine Institute, Rinville, Oranmore, Co., Galway, Ireland; 4 UCD-Centre for Food Safety, School of Public Health, Physiotherapy & Sports Science, Belfield, Dublin, 4 Ireland

**Keywords:** Berries, Detection, Foodborne virus, Risk assessment, Sapovirus

## Abstract

Soft fruits are at particular risk of contamination with enteric viruses such as Hepatitis A virus (HAV), Hepatitis E Virus (HEV), Norovirus (NoV), Human Adenovirus (HAdV) and Sapovirus (SaV). The aim of this study was to investigate, for the first time, the presence of these biological agents in ready to eat (RTE) berries at point of retail in Ireland. A sampling strategy was designed in which RTE fresh and frozen strawberries and raspberries were purchased from five retailers between May and October 2018. Reverse Transcriptase Polymerase Chain Reaction (RT-qPCR) assays for HEV RNA, Nov RNA, SaV RNA, and human Adenovirus species F DNA (HAdV-F) were performed on 239 samples (25g portions). Viral nucleic acid was present in 6.7% (*n* = 16) of samples tested as follows: HAV RNA (*n* = 5), HAdV-F DNA (*n* = 5), HEV RNA (*n* = 3) and NoV GII RNA (*n *= 3). Sapovirus RNA was not detected in any product. No significant differences were found between berry type, fresh/frozen status, or supermarket source. This study suggests a risk that exists across all retail outlets however only low levels of nucleic acid ranging from 0 to 16 genome copies/g were present. Although these findings may reflect non-viable/non-infectious virus the continued provision of risk mitigation advice to consumers is warranted and further work is required to ensure control measures to reduce contamination are implemented and enforced.

## Introduction

In Ireland, 33% of adults report consuming 5 portions or more of fruit and vegetables each day, the highest among EU member states (OECD & European Union, 2022). The popularity of fresh and frozen berry products in particular, drives a year-round demand which is met by national production and the import market. The health benefits of eating berries are well described: however, they are also acknowledged to be at risk of contamination with enteric viruses such as Human Norovirus (NoV), Hepatitis A (HAV), Hepatitis E (HEV), Sapovirus (SaV) and Human Adenoviruses (H-AdV) (Chatonnat et al., 2023; Kobayashi et al., 2012; Maunula et al., 2013).

Although NoV, SaV and H-AdV-F generally cause self-limiting gastrointestinal illness, HAV can cause significant morbidity and mortality (Elmahdy et al., 2022). Similarly, the natural course of HEV infection is asymptomatic in the majority but severe illness including hepatitis, neurological disease, and chronic infection are reported (EFSA et al., 2017).

Guidance exists to support microbiological control measures for the production of fresh fruit and vegetables for consumption: however, the contamination of soft fruit can occur at multiple points along the production chain. Pre-harvest risks may be due to contact with contaminated soil or irrigation water. During harvest and post-harvest, contamination can occur due to unhygienic practices by infected food handlers or by contact with contaminated surfaces, water, or other produce (EFSA, 2011). As viral pathogens can persist in these products for long periods of time, and often have a low infectious dose even minor levels of contamination can be a risk to public health (Bosch et al., 2018). Available evidence suggests that freezing does not totally eliminate or inactivate enteric viruses such as HAV or NoV so that berries which are freeze dried or frozen can act as sources of infection for outbreaks which may be geographically and temporally dispersed (Butot et al., 2008; NASHERI et al., 2020).

In recent years, significant advances in detection methodologies for viruses in food have culminated in the publication of International Technical Standards to test for NoV RNA and HAV RNA in fruits, vegetables, water and bivalve molluscs. (International Standards Organization, 2017). Additional RT-qPCR methods can be applied to extracted products to investigate for the presence of HEV RNA,SaV RNA and HAdV DNA (Garson et al., 2012; Jothikumar et al., 2006; Oka et al., 2006). Although such methods cannot distinguish between infectious and nonviable virus, they are nonetheless important methods to assess the effectiveness of control measures in food production. The detection of H-AdV DNA in particular, has been highlighted as a useful indicator of faecal contamination in light of the virus’ human host specificity, prevalence, environmental persistence and resistance to UV radiation (Marti et al., 2017; Li et al., 2018).

Utilizing methodologies based on existing ISO standards the aim of this study was to investigate for the first time the presence of HEV RNA, HAV RNA, NoV RNA SaV RNA and HAdV-F DNA in fresh and frozen Strawberries and Raspberries at the point of retail in Ireland.

## Materials and Methods

### Sampling

As this was the first study to investigate for the presence of viral RNA in our country a number of assumptions were made for the sampling strategy. Consumption of fresh fruit, including nationally produced berries is known to be highest during summer months so the survey was carried out between May and October 2018. Samples were purchased on a weekly basis from the top five Irish retailers, selected based on their grocery market share, three large supermarket chains and two discount supermarkets. Over the 25-week period, 240 samples comprising fresh strawberry punnets (*n* = 63), fresh raspberry punnets (*n* = 60), frozen strawberries (*n* = 57) and frozen raspberries (*n* = 60) were purchased. Data collected for each sample included a description of the product and the date of purchase. Details of the vendor and country of origin were not recorded. Samples were transported to the laboratory in a non-temperature monitored cool bag before freezing and stored at − 20 °C prior to testing.

### Viral Recovery from Fresh and Frozen Berries

This protocol was as described for detection of NoV RNA and HAV RNA from soft fruit in the ISO technical specification ISO 15216-1:2017(International Standards Organization, 2017). Briefly, each sample (25g) was placed in a 400 mL polypropylene mesh filter bag and 40mL of buffer (100 mM Tris–HCl, 50 mM glycine, 1% beef extract, pH 9.5) (Sigma Aldrich) was added along with 10 µl of internal process control consisting of Mengovirus (MeV) strain MC0 (ATCC-1957) and 300 µl (30units) of Pectinase (*Aspergillus aculeatus* Sigma Aldrich, P2611). The bag was rocked for 20 min before the supernatant was removed via the filter compartment of the bag and centrifuged at 10,000 × g for 30 min at 4 °C to pellet the food debris. The pH of the decanted supernatant was adjusted to 7.0 + / − 0.2 with the addition of 5 N HCl. 0.25 volumes of 5 × 500/1 poly-ethylene glycol (PEG) 8000 (Sigma-Aldrich), and 1.5 M NaCl, and was added to the supernatant and incubated 1 h at 4°C. Viruses were concentrated by centrifugation at 10,000 × g for 30 min at 4 °C. The supernatant was discarded, and an additional centrifugation was performed at 10,000 × g for 5min at 4 °C to compact the pellet. The pellet was suspended in 500 µl of PBS and vortexed with 500 µl of 1:1 chloroform: butanol (v/v). The suspension was then incubated for 5 min at room temperature, and centrifuged at 10,000 × g for 5min at 5 °C. The upper aqueous phase containing viruses was directly processed using the nucleic acid extraction procedure. A negative process control was also prepared along with each batch of samples.

### Nucleic Acid Extraction

All nucleic acid was extracted on the same day as viral recovery from the food product to reduce any degradative effects or acidity from the extraction process. A volume of 1ml of each sample along with a negative extraction control was extracted using Nuclisens easyMAG automated extraction system (Biomerieux). 100 μl of nucleic acid was eluted. A further cleaning step to reduce PCR inhibition was carried out using OneStep-96 PCR Inhibitor Removal kit (Zymo Research, D6035). In brief, 150 μl of the assay’s Prep- Solution was added to Silicon-A™-HRC Plate, incubated for 5 min, before centrifuging the plate at exactly 3,500 × g for 5 min. 100μl of eluted RNA was then added to the plate and centrifuged at 3,500 × g for 3 min. The filtered RNA was stored at – 80 °C for further analysis.

### RT-qPCR for NoV RNA, HAV RNA, SaV RNA, HEV RNA, MeV RNA, and HAdV-F DNA Detection

One-step Real -time PCR (RT-qPCR) amplifications were performed in duplicate on Applied Biosystems 7500 Fast Real-Time PCR System. Reactions were performed in a 25 μL reaction mixture containing 12.5 μL of SuperScript III Platinum One Step Quantitative RT-qPCR reaction mix (Invitrogen), and 0.8 µl of Superscript III/Platinum Taq Mix. The following concentrations for primers and probes as outlined in the ISO technical specification (ISO 15216-1:2017), 500 nM (HAV, NoV GI, and NoV GII) of forward primer, 900nM (HAV, NoV GI, and NoV GII) of anti-sense primer, 250 nM of probe for all viral targets and 5 μL of RNA extract. The primers and probes for the detection of SaV RNA were made up to the following concentrations: 800 nm for forward and reverse primers and 50 nm for each of the probes (Oka et al., 2006). Positive controls containing RNA extracted from virus suspensions and a negative control containing all the reagents except the RNA template were included with each set of reaction mixtures. The one-step RT-qPCR programme involved a 15 min reverse-transcription of RNA at 50 °C, followed by a 2 min denaturation step at 95 °C, and finally 45 cycles of 15s at 95 °C, 30 s min at 56 °C. Fluorescence was recorded by the instrument at the end of the elongation steps (1 min at 65 °C) for each amplification cycle.

Detection of HEV RNA was carried out using 5 µl of sample RNA together with 12.5 μL Superscript^™^ III Platinum^®^ One-Step qRT-PCR reaction mix (Invitrogen), 0.5 µl of Superscript III/Platinum Taq Mix was added to the following concentrations for primers and probe: 200 nM for forward primer 200 nM for reverse primer (Metabion), and 200 nM for probe (Life Technologies) (Garson et al., 2012; Jothikumar et al., 2006). RT-qPCR was carried out at 50 °C for 15min, followed by inactivation for 2 min at 95 °C and 45 cycles of 15 secs at 95 °C and 34secs at 60 °C.

A qualitative assay to detect HAdV-F was carried out using 5 μL of DNA extract together with 12.5 μL of SuperScript III Platinum One Step Quantitative RT-qPCR reaction mix (Invitrogen), 0.8 µl of Superscript III/Platinum Taq Mix and 1 µl of 25 × detection mix containing 600nM of forward primer, 600nM of anti-sense primer, 100 nM of probe (Tiemessen & Nel, 1996). The one-step RT-qPCR programme involved a 15 min reverse-transcription of RNA at 50 °C, followed by a 2 min denaturation step at 95 °C, and finally 45 cycles of 15 s at 95 °C, 30s at 60 °C. All samples were characterised by a corresponding Cq value. Negative samples gave no Cq value.

A standard curve for NoV GI RNA, NoV GII RNA, and HAV RNA was generated by cloning NoV GI (90 nucleotides) and GII (95 nucleotides), and HAV RNA inserts into pGEM-3Zf (+) vectors for the production of dsDNA linear plasmids. A SaV RNA (104 nucleotide) insert was cloned into a pUC57 vector, this allowed for the preparation of RNA transcripts of the same sequence, and were supplied by The Marine Institute, Ireland. HEV RNA standard curve was prepared using synthetic dsDNA (Amp Tec, Germany). All standards were diluted 10^1^ − 10^5^ copies/µl and tested in duplicate on the PCR plate. Curves with *r*^2^ values of > 0.980, or where the slope was between − 3.10 and − 3.60 was used for calculations. The average concentration of the sample replicates was used to determine quantification.

The theoretical limit of detection”(tLOD’) and theoretical limit of quantification (tLOQ) were determined for NoV GI RNA, NoV GII RNA, HAV RNA, SaV RNA, and HEV RNA using the modified method of Armbruster and Pry (2008) and Bustin et al. (2009). For each assay, serial dilutions (6 dilutions × 10 replicates) of the standard were analysed. The lowest dilution with a standard deviation (σ_CtLoD_) of < 1 and detection > 95% was used to calculate the average CtLoD. The CtLOQ was calculated as the CtLOD minus 2(*σ*_CtLOD_). The tLOD and tLOQ were calculated by subtracting the y-intercept and dividing by the slope.

To assess RT-qPCR inhibition an external amplification control RNA (EC RNA) was added to each sample. The Cq value of sample RNA+EC RNA should be < 2,00 greater than the Cq value of the water+EC RNA well, If the Cq value > 2,00 greater than the Cq value of the water+EC RNA well, results are invalid, and the sample should be retested.

### Extraction Efficiency

Extraction efficiency was calculated using the Cq values obtained from each test sample RNA well by reference to the mengo virus RNA standard curve. Mengo virus recovery = 10^(ΔCq/slope) × 100%where Δcq = Cq value (sample RNA)—Cq value (undiluted process control virus RNA) A sample producing the same Cq value as undiluted mengo virus RNA has an extraction efficiency of 100%. Where the extraction efficiency was < 1% sample results were considered invalid and are retested**.**

### Statistical Analysis

#### Statistical Methods

Cq data was transferred manually to Microsoft Excel^®^ (Microsoft corporation, 2013), for calculation of concentrations. Final data was exported to csv for analysis and plotting in the R statistical language, version 4.1.1 (R Core Team, 2017), using the IDE RStudio 2021.09.0 and the libraries “dplyr”, “tidyr”, “ggplot2”, and “binom”.

#### Prevalence

Prevalence’s were calculated by Wilson score interval (Brown et al., 2001) using the R package “binom”. Calculations for all samples across the sampling period, and within subgroups, were based on the three factors of (1) berry type (raspberry/strawberry), (2) fresh/frozen status, and (3) retailer (1–5), as well as combinations of the three. Logistic regression was used to assess the relationship between those three factors and the presence or absence of viral contamination, for each virus. Forward stepwise logistic regression was then used to identify possible relevant factor interactions. Starting from an intercept only model, at each step the next most significant factor or interaction, based on p-values, was added as a predictor, until no decrease in AIC could be observed.

#### Concentration

Where positive virus results could be quantified, the distributions of these results were analysed separately from the non-detects. Mean concentrations were calculated for each quantifiable target virus both as an aggregate across all samples and within the subgroups defined by the three factors. Standard deviations were also calculated, where groups and viruses had more than one quantifiable result. Multiple linear regression was used to assess the influence of each factor on the final mean concentration for each virus. Forward stepwise linear regression was then used to identify the significant factor interactions, if any.

#### Seasonal Prevalence

For each product type (berry and fresh/frozen status), the trend in detection rate was plotted over time to illustrate any potential seasonal exposure effects for the public. Logistic regression was used to assess the influence of month on the proportion of positive samples, for viruses individually and for all viruses in aggregate.

## Results

In total 240 berry samples were tested using the modified version of the ISO standard ISO 15216-1:2017. RT-qPCR for HAV RNA, HEV RNA, NoV GI RNA, NoV GII RNA, SaV RNA, and RT-PCR for HAdV-F DNA was performed on all samples with an extraction efficiency > 1% (*n* = 239). All quality controls passed and no RT-PCR inhibition was observed. A summary of results is described in Table 1. Viral nucleic acid was present in 6.7% (*n* = 16). The most frequently detected nucleic acids were HAV RNA (*n* = 5) and HAdV-F DNA (*n* = 5). In addition, HEV RNA (*n* = 3) and NoV GII RNA (*n* = 3) were detected, but SaV RNA and NoV GI RNA was not found in any sample. HAV RNA was found at levels below the limit of detection of the standard assays suggesting very low levels of contamination. The product type with the highest level of contamination was the frozen strawberries (Table 1) One frozen strawberry product out of the sample set tested positive for both HEV RNA and HAdV-F DNA,

**Table 1:** Summary of viral detection in fresh and frozen berry samples

Viral pathogen	tLOD genome copies/g	tLOQ genome copies/g	No. of positive detections	Matrix	Average Cq value	Result genome copies/g
HAV	2.14	2.73	5	Fresh Strawberry	37.7	0.3
				Fresh Raspberry	37.0	0.5
				Fresh Strawberry	38.2	0.3
				Fresh Raspberry	37.4	0.6
				Frozen Strawberry	37.8	0.4
HEV	1.1	1.63	3	Frozen Strawberry	39.2	0.5
				Frozen Strawberry	36.8	2.0
				Fresh Raspberry	33.7	16.0
NoV GII	1.7	2.3	3	Frozen Strawberry	37.9	0.9
				Frozen Raspberry	36.5	3.6*
				Frozen Strawberry	36.5	7.8
HAdV-f	n/a	n/a	5	Frozen Strawberry	40.9	n/a
				Frozen Strawberry	42.9	n/a
				Frozen Strawberry	37.5	n/a
				Frozen Raspberry	37.8	n/a
				Frozen Raspberry	44.4	n/a
NoV GI	1.85	2.43		n/a		n/a
SaV	2.04	2.63		n/a		n/a

^*^Only one isolate positive when tested in duplicate

No significant differences were found with logistic regression between berries, fresh/frozen status, supermarket, or any combinations. In addition, a multiple regression was fit to the mean concentrations, using virus, berry, fresh/frozen status and supermarket as predictors, including interaction effects. No significant associations were found to distinguish between any factor or combination of factors. Forward stepwise regression, introducing factors and interaction effects one at a time, also did not find any significant predictors.

**Fig. 1:**
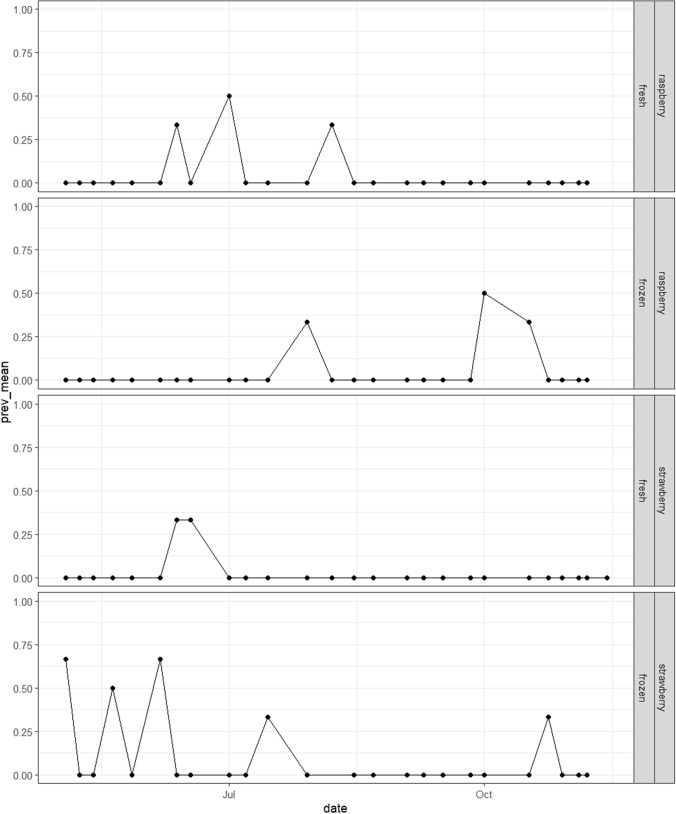
Weekly trend (by collection date) in proportion of samples testing positive for at least one virus target, separated by product category

The trend in detection rates over time for samples containing one or more virus targets is shown in Fig. 1. A multiple regression model was fitted to the number of total positive counts per month, to assess the significance in difference by month or by product. No significant differences were found between months, with or without product types as predictor. A one-way ANOVA model also found no significant difference between months as factor levels.

## Discussion

This study describes the first retail survey of foodborne viruses in fresh and frozen berries in Ireland in which viral RNA was detected in 6.7% (*n* = 16) samples. It is notable that HAV was the most frequently detected viral RNA in this study (*n* = 5) albeit at very low levels. Ireland is considered to be a country with a low incidence of HAV infection (case notification rate of 0.7/100,000 population (HPSC, 2017). Although source attribution in autochthonous Irish cases can be difficult, foodborne transmission likely contributes as HAV is an extremely resilient virus and foodborne outbreaks are well described (Lemon et al., 2018) The risk of HAV in contaminated frozen berries for Irish consumers was previously highlighted in 2013, when HAV cases from a European outbreak associated with frozen berries were identified in Ireland by molecular sequencing of clinical isolates. At this time however, there was no facility to expand the investigation into food products (EFSA, 2014; Fitzgerald et al., 2014). In our study 4 out of the 5 positive samples in which RNA was detected were fresh berries. The risk of HAV infection associated with berry consumption is well described but a review by Bozkurt et al. identified only 5/22 reported outbreaks of HAV were linked to consumption of fresh products (2021). Although high rates of HAV RNA detection (48% of fresh berry products) have recently been reported in Egypt (Elmahdy et al., 2022) the overall rate of HAV detection in this study (2%) is similar to reports from Italy, Argentina, Czech Republic and Australia where the detection of HAV in fresh and frozen berry products range from 0% to 2% (Dziedzinska et al., 2018; Macori et al., 2018; Oteiza et al., 2022; Pavoni et al., 2022; Torok et al., 2019).

In addition to HAV RNA, one of the samples of frozen strawberries was dual positive for HAdV Subtype F DNA. Detection of HAdV has previously been utilised as a marker of faecal contamination of fresh produce as HAdV are robust nonenveloped DNA viruses and large viral loads can be shed in the faeces of an infected individual (Li et al., 2018; Maunula et al., 2013). Although Human adenoviruses can cause a wide spectrum of illness gastrointestinal symptoms are predominantly associated with HAdV subtype F the target of this study (Lion, 2014). In total five samples were positive for HAdV-subtype F suggesting that further work is required to enforce control measures in soft fruit production and the potential for HAdV gastrointestinal infections to be transmitted through these products.

In recent years there has been an increased focus on Hepatitis E as a foodborne pathogen with consumption of contaminated pork and pork products considered to be a key driver of Hepatitis E infection in Europe (EFSA et al., 2017; SAID et al., 2014). However, the detection of HEV RNA in three frozen berry samples (1.3%) at different timepoints in our study adds further evidence that products other than that those of porcine origin may act as sources of infection (Treagus et al., 2021). Although the infectious dose of HEV is unknown it is notable that of the detectable RNA found in our study HEV was responsible for the largest viral load (400 copies/25 g). HEV contamination of frozen raspberries has previously been reported by (Maunula et al., 2013) and HEV RNA has been detected in field grown strawberries suspected to be irrigated with contaminated water (Brassard et al., 2012).

NoV is an extremely stable virus making it a very successful pathogen associated with food borne illness (Amarasiri & Sano, 2019). In addition, just one infectious NoV particle has been shown to cause infection in human volunteer studies (Teunis et al., 2008). The results from this study identified 1.3% (*n* = 3/239) of berries tested had detectable NoV GII RNA, two of which were above the limit of detection of the assay yielding 90 copies/25 g and 195 copies/25 g respectively. Though it is traditionally thought NoV GI is more commonly associated with food and water borne outbreaks no NoV GI strains were found in this study (da Silva et al., 2007),

To the best of our knowledge this retail survey is the first look at the risk of SaV to the consumer in fresh and frozen berries. SaV belongs along with NoV to the family Caliciviridae, and is known to cause the same symptoms such as diarrhoea, and vomiting (Franck et al., 2015). SaV outbreaks linked to foodborne transmission are well recognised, in particular because asymptomatic workers may shed high viral loads for prolonged periods (EFSA, 2021) (Yoshida et al., 2009). One of the largest SaV outbreaks reported affected 665 individuals in Japan where an epidemiological investigation pointed to contaminated box lunches prepared by food handlers who were shedding the virus (Kobayashi et al., 2012). In this study despite the external control RNA (ECRNA) amplifying in all samples tested, SaV was not detected in any product.

Sample collection for this study was carried out during May to October, the time period of national production, peak consumption and thus availability of fresh soft fruit in Ireland. Trends in viral detection per product type and over the season were investigated but given the low number of total positives (16 out of 239 samples), statistical significance could not be established for any predictor. Of the viral pathogens isolated from the soft fruit purchased at retail, 69% (*n* = 11) of the products with detectable viral RNA were frozen berries. A review by Bozkurt et al., (2021) reported that frozen berries were implicated in fifty documented outbreaks of human NoV (*n* = 36) and HAV (*n* = 14) (2021). The increased risk of contamination from frozen berries may be involved on a larger scale due to poor processing practices in the factory and the introduction of many berries from multiple country of origin packed together (Tavoschi et al., 2015). Frozen berries are considered a particular risk to consumers as given the long shelf-life one batch of contaminated fruit may act as a point of infection for multiple outbreaks over prolonged timed periods (EFSA, 2014). Our findings support the need for increased promotion of existing public health messaging to boil frozen berries for one minute before consumption (FSAI, 2017).

Aside from the constraints in statistical analysis posed by the low number of positive results one of the key limitations of this study is that it was not possible to determine if detection of nucleic acid reflected viable virus which could potentially cause infection. The development of viral integrity assays and more robust cell culture systems may help to address this in future work (Leifels et al., 2020) In addition, due to low levels of viral RNA recovered sequence analysis could not be performed, so no investigation with available HEV, HAV and NoV isolates from clinical samples could be undertaken.

In conclusion, this is the first comprehensive study of foodborne viruses in fresh and frozen berries in Ireland. While a burden of viral contamination was observed at the point of retail, further work is required to quantitate the risk to public health. Notwithstanding these limitations our findings have implications for Irish and international fruit producers highlighting the need for development and enforcement of control strategies to prevent contamination and cross contamination of soft fruit for human consumption.
